# Supplementary material for: Graph Structured Neural Networks for Perturbation Biology
Source: bioRxiv. 2024 Feb 29:2024.02.28.582164. Preprint. [Version 1] doi: 10.1101/2024.02.28.582164 (PMC10925270; doi:10.1101/2024.02.28.582164)
Supplement: Supplement 1 [file NIHPP2024.02.28.582164v1-supplement-1.pdf]

bioRxiv preprint doi: https://doi.org/10.1101/2024.02.28.582164; this version posted February 29, 2024. The copyright holder for this preprint (which was not certified by peer review) is the author/funder, who has granted bioRxiv a license to display the preprint in perpetuity. It is made available under aCC-BY 4.0 International license.

9 Supplementary

9.1 Experiment Details

Each experiment is described by a set of hyperparameters that specify the nodes (drugs, proteins, RNA & LINCS genes), cell lines, and observations that will be included. One of the key parameters is the choice of proteins to be included in the biological graph on which the GSNN will operate. Ideally, we would select a subset of proteins that are relevant to a certain drug or set of drugs; however, inferring which proteins are involved in a given drug response is a challenging task. In lieu of identifying proteins that are relevant to the drug response, we select proteins based on their involvement with specific biological processes or pathways and manually select the subset of pathways that we believe are likely to be involved in the drug response. In experiments 1-3, we first select a set *primary* Reactome pathways and then manually comb through each primary pathway to identify *linked* pathways[17]. To avoid exceptionally large protein sets, we use our discretion to select *linked* pathways which we believe are relevant to the *primary* pathway. For instance, we generally avoided including pathways that are unlikely to be well modeled by the GSNN premise of cellular signaling such as DNA damage response[18]. We also did not include disease pathways, which Reactome has designated as separate pathways; therefore, all specified pathways should be considered canonical and healthy signaling processes. The experiment pathway details are shown in Table 8. Although each experiment has a unique set of *primary* pathways, there are many included *linked* pathways which are shared by all three experiments. Additionally, many proteins have multiple roles in several pathways. This overlap in pathways and protein roles means that even distinct experiment pathway parameters may result in relatively similar biological networks. Figure 10 shows the overlapping entities between experiments 1-3. Of note, while most elements have substantial overlap between each experiment, the protein-space[19] is relatively distinct between each experiment.

[17] Pathways that are not subpathways but are referenced within a pathway, e.g., pathway A -> activates -> pathway B but pathway B is not a subpathway of A)

[18] We are concerned that DNA damage drugs will not be well represented by the drug-target premise

[19] all proteins included in the biological network

bioRxiv preprint doi: https://doi.org/10.1101/2024.02.28.582164; this version posted February 29, 2024. The copyright holder for this preprint (which was not certified by peer review) is the author/funder, who has granted bioRxiv a license to display the preprint in perpetuity. It is made available under aCC-BY 4.0 International license.

Reactome ID	Description	Size
**R-HSA-177929**	**Signaling by EGFR**	**52**
R-HSA-1489509	DAG and IP3 signaling	41
R-HSA-1257604	PIP3 activates AKT signaling	282
R-HSA-5673001	RAF/MAP kinase cascade	292
**R-HSA-1227986**	**Signaling by ERBB2**	**56**
R-HSA-109606	Intrinsic Pathway for Apoptosis	55
R-HSA-6806003	Regulation of TP53 Expression and Degradation	37
R-HSA-202131	Metabolism of nitric oxide: NOS3 activation and regulation	26
R-HSA-6807070	PTEN Regulation	139

(a) Exp. 1

Reactome ID	Description	Size
**R-HSA-73887**	**Death Receptor Signaling**	**161**
R-HSA-75157	FasL/ CD95L signaling	5
R-HSA-140534	Caspase activation via Death Receptors in the presence of ligand	19
R-HSA-75158	TRAIL signaling	8
R-HSA-75893	TNF signaling	61
R-HSA-5218859	Regulated Necrosis	62
R-HSA-5213460	RIPK1-mediated regulated necrosis	35
R-HSA-5620971	Pyroptosis	27
R-HSA-109606	Intrinsic Pathway for Apoptosis	55
R-HSA-446652	Interleukin-1 family signaling	155
R-HSA-5686938	Regulation of TLR by endogenous ligand	21
R-HSA-193704	p75 NTR receptor-mediated signalling	99
R-HSA-187037	Signaling by NTRK1 (TRKA)	117
R-HSA-5673001	RAF/MAP kinase cascade	292
R-HSA-1257604	PIP3 activates AKT signaling	282
R-HSA-9031628	NGF-stimulated transcription	39
R-HSA-1489509	DAG and IP3 signaling	41

(b) Exp. 2

Reactome ID	Description	Size
**R-HSA-201556**	**Signaling by ALK**	**28**
R-HSA-1257604	PIP3 activates AKT signaling	282
R-HSA-165159	MTOR signalling	41
R-HSA-380972	Energy dependent regulation of mTOR by LKB1-AMPK	29
R-HSA-6807070	PTEN Regulation	139
R-HSA-109606	Intrinsic Pathway for Apoptosis	55
R-HSA-202131	Metabolism of nitric oxide: NOS3 activation and regulation	14
R-HSA-6806003	Regulation of TP53 Expression and Degradation	37
R-HSA-6804756	Regulation of TP53 Activity through Phosphorylation	92
R-HSA-5693606	DNA Double Strand Break Response	61
R-HSA-5673001	RAF/MAP kinase cascade	292
R-HSA-1489509	DAG and IP3 signaling	41

(c) Exp. 3

Table 8: (a-c) The pathways that were used in each experiment to specify the proteins included in the GSNN input graph. Bold text indicates the initial pathway choice from which all other pathways were "linked." Pathway size refers to the number of proteins in each reactome pathway and may not reflect the exact number of proteins included in the resulting biological network.

bioRxiv preprint doi: https://doi.org/10.1101/2024.02.28.582164; this version posted February 29, 2024. The copyright holder for this preprint (which was not certified by peer review) is the author/funder, who has granted bioRxiv a license to display the preprint in perpetuity. It is made available under aCC-BY 4.0 International license.

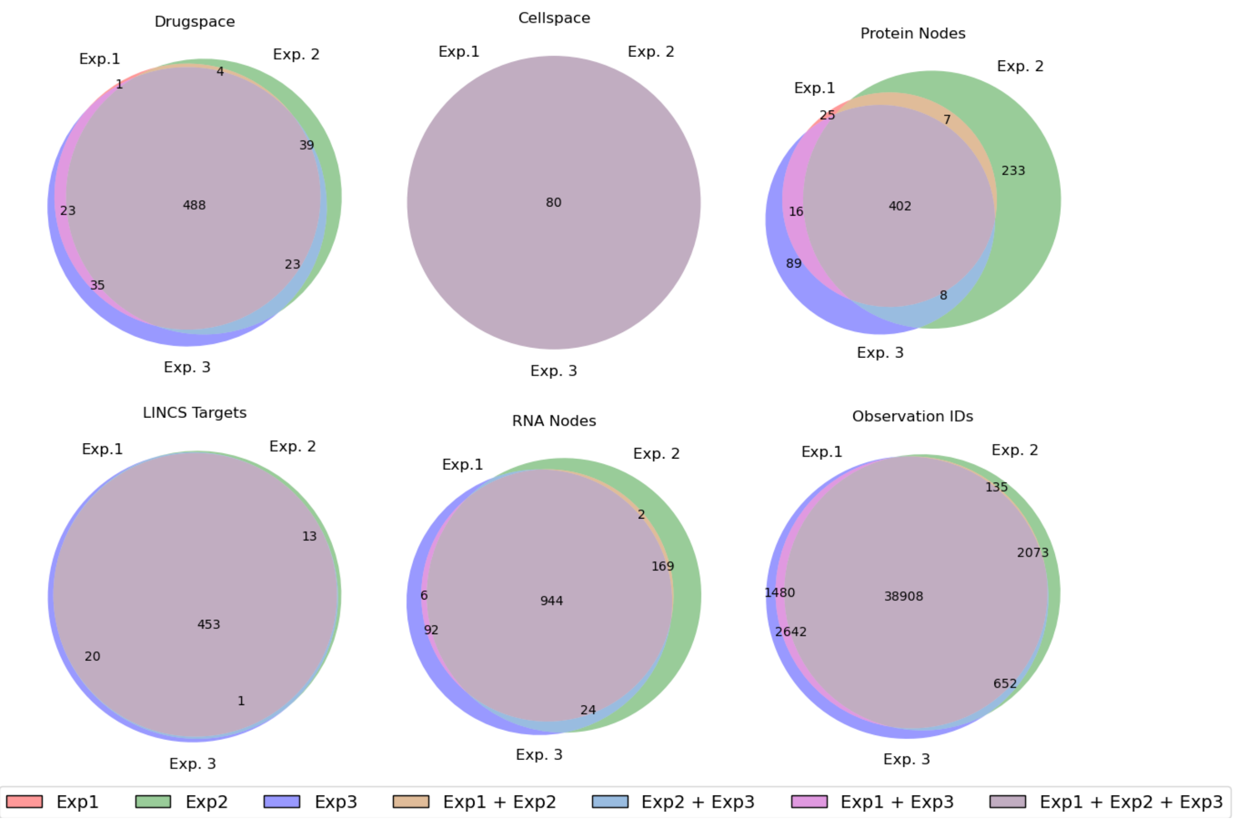

Figure 10: The overlapping elements of each experiment. Most of the targets, nodes and drugs are shared across all three experiments; however, there are distinct protein subsets for each of the three experiments.

9.2 Number of model parameters: GSNN vs. NN

In table 9 we report the number of GSNN and NN parameters in the best-performing models of each experiment. Across all three experiments, the best GSNN models from each fold had more trainable parameters than the best NN model from that fold. This may be indicative of the GSNN model being less prone to over-fitting. Another explanation is that, due to the GSNN biological graph structure, there are likely to be many function nodes that are rarely involved in prediction logic or impact only a few targets (i.e., only a few LINCS nodes are descendants) and therefore the functional set of parameters may not be well represented by the total number of trainable parameters. In other words, prior knowledge may lead to some function nodes being effectively spurious or underutilized, and therefore the direct parameter comparison should be interpreted with caution.

Table 9: Number of trainable parameters of the GSNN and NN algorithms used in experiments 1-3 (median of best models from each MCCV fold). Percent change is calculated as $\frac{N_{gsnn}-N_{NN}}{N_{nn}}$, where N is the median number of algorithm parameters

EXP. ID	Num. GSNN params	Num. NN params	Percent Change
exp1	6.56e+06	2.23e+06	193.8 %
exp2	7.85e+06	5.68e+06	38.2 %
exp3	6.70e+06	5.3e+06	26.5 %
AVG.			86.2 %

9.3 Computational Complexity of the GSNN method

The GSNN algorithm takes significantly longer to train due to being a particularly deep architecture and due to it's use of sparse matrix operations. Table 10 reports the average training times for each algorithm. Specifically, the

bioRxiv preprint doi: https://doi.org/10.1101/2024.02.28.582164; this version posted February 29, 2024. The copyright holder for this preprint (which was not certified by peer review) is the author/funder, who has granted bioRxiv a license to display the preprint in perpetuity. It is made available under aCC-BY 4.0 International license.

GSNN algorithm requires between 3-15 times as much training time as the alternative algorithms tested (NN, GNN). Of note, however, are the training curves shown in Figure 11 that compare the validation performance by epoch for representative GSNN and NN models; The GSNN validation performance increases markedly faster, achieving approximately the maximum NN performance in the first 20 epochs. This aspect of the training dynamics may suggest that the GSNN algorithm can be trained with fewer epochs, which would markedly reduce the compute requirements.

Table 10: Average training time of each algorithm (reported in minutes). Note: GSNN and GNN were trained on GPUs whereas the NNs were trained on CPU only.

EXP.	GSNN	GNN	NN	GSNN / NN	GSNN / GNN
exp1	419.8	105.0	29.0	14.5	4.0
exp2	493.3	138.4	32.4	15.2	3.6
exp3	460.8	133.9	35.9	12.9	3.4

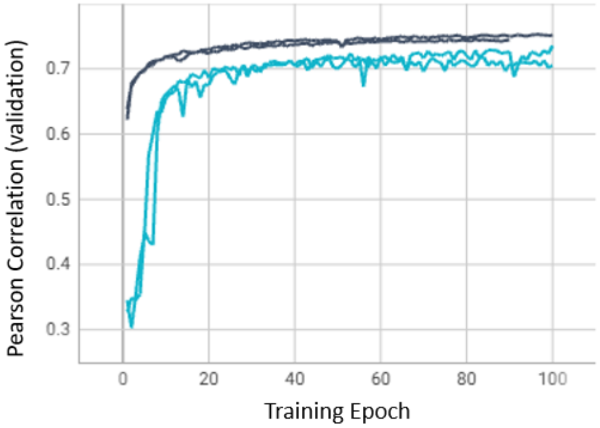

Figure 11: Representative training curves from experiment 1 (EGFR + ERBB2 signaling). Dark Gray/Blue indicates the GSNN training curves, light blue are NN training curves.

9.4 Effect of Layer Depth on GSNN performance

The GSNN algorithm passes information during sequential *layers* allowing information to diffuse through the network up to the number of layers L in the model. Cell signaling often involves many entities in many sequential interactions as well as feedback loops that may alter behavior. Due to this trait, deeper networks may be more representative of the underlying biology and therefore more accurate. To test this, we compare the performance of GSNN algorithms with different number of layers (L=10,20). Figure 12 shows the results and suggests that 20-layer GSNNs have a small improvement in performance compared to 10-layer GSNNs. Notably, training deeper neural networks also introduces more parameters, greater memory complexity, and longer training times. It is critical, therefore, that the choice of GSNN layers be tailored to the available hardware and training budget. Improvements in time complexity of the GSNN algorithm may enable deeper and more accurate models.

bioRxiv preprint doi: https://doi.org/10.1101/2024.02.28.582164; this version posted February 29, 2024. The copyright holder for this preprint (which was not certified by peer review) is the author/funder, who has granted bioRxiv a license to display the preprint in perpetuity. It is made available under aCC-BY 4.0 International license.

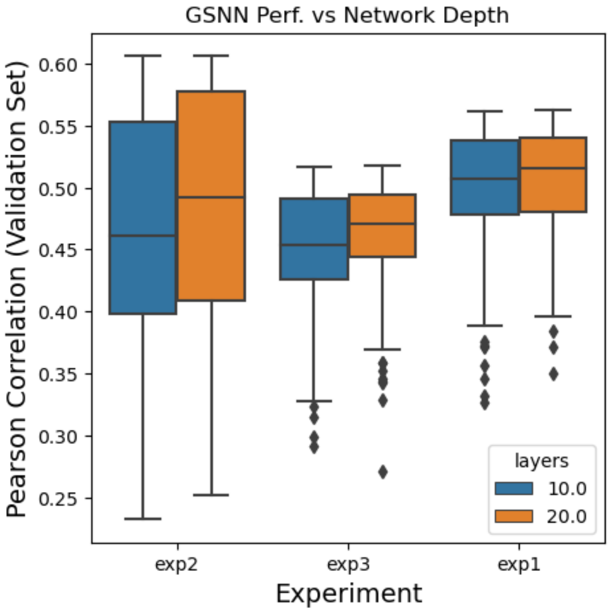

Figure 12: The performance of GSNN algorithms in experiments 1-3 compared by the number of layers (L) hyper-parameter.
